# Diazines and Triazines as Building Blocks in Ligands for Metal-Mediated Catalytic Transformations

**DOI:** 10.1021/acsorginorgau.3c00048

**Published:** 2023-10-23

**Authors:** Julianna S. Doll, Felix J. Becker, Dragoş-Adrian Roşca

**Affiliations:** Anorganisch-Chemisches Institut, Universität Heidelberg, Im Neuenheimer Feld 276, 69120 Heidelberg, Germany

**Keywords:** Ligand design, pyrazine, pyrimidine, triazine, metal−ligand cooperativity, redox-active ligands, mechanistic studies, catalysis

## Abstract

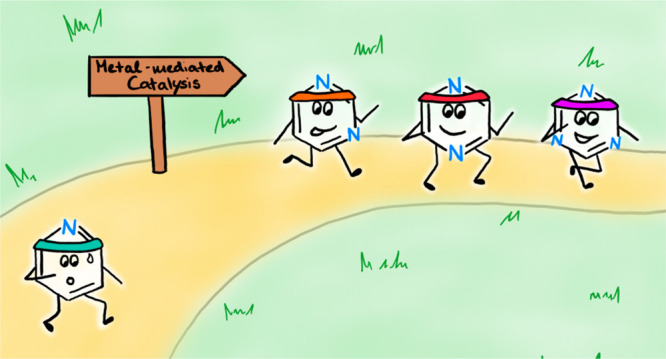

Pyridine is a ubiquitous building block for the design of very diverse ligand platforms, many of which have become indispensable for catalytic transformations. Nevertheless, the isosteric pyrazine, pyrimidine, and triazine congeners have enjoyed thus far a less privileged role in ligand design. In this review, several applications of such fragments in the design of new catalysts are presented. In a significant number of cases described, diazine- and triazine-based ligands either outperform their pyridine congeners or offer alternative catalytic pathways which enable new reactivities. The potential opportunities unlocked by using these building blocks in ligand design are discussed, and the origin of the enhanced catalytic activity is highlighted where mechanistic studies are available.

## Introduction

### Pyridines as Ligands in Coordination Chemistry–A Brief Overview

**Figure 1:**
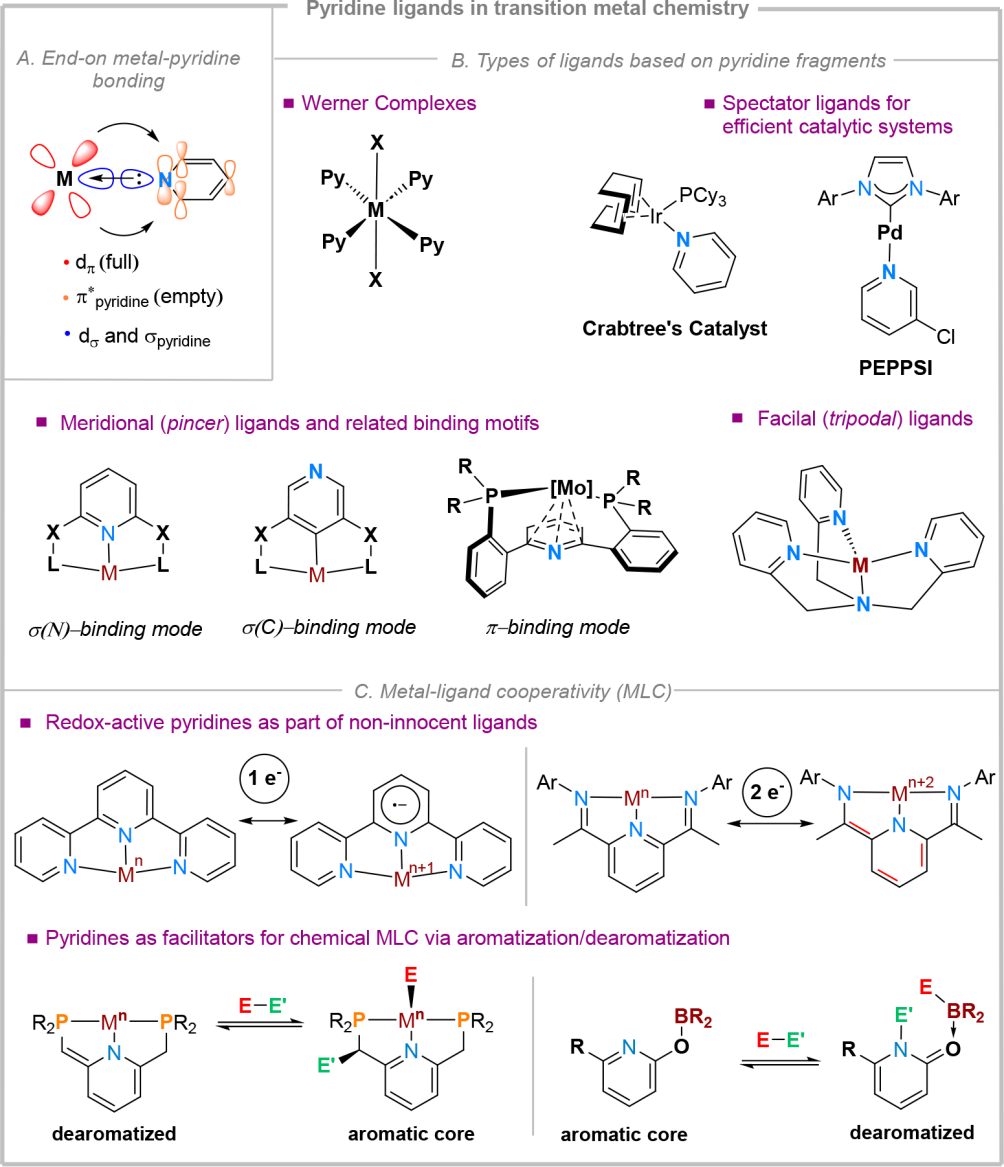
Brief overview of role and binding modes of pyridine ligands in transition metal chemistry.

Pyridine cores are fundamental building blocks for constructing a large variation of ligand scaffolds in coordination chemistry. Depending on the envisaged application, these ligands have found a wide range of roles and can bind, essentially, all the stable metals of the periodic table. Among the multitude of applications, they are frequently used in the construction of new catalysts or in the stabilization of highly reactive intermediates, to name a few. Traditionally, pyridine ligands frequently function as spectator (ancillary) ligands (i.e., Werner complexes, Figure 1) and are essential components of established catalytic systems (*e.g*., the Crabtree catalyst for hydrogenation or PEPPSI catalyst for challenging cross coupling reactions). Typically, three coordination modes can be considered *(i)* end-on coordination, through the N atom lone pair, or *(ii)* side-on, through the ring π-electrons (analogous to the η^2−6^ coordination modes of arenes), and *(iii)* end-on coordination, through one of the carbon atoms. In the latter two cases, the N atom remains available for further functionalization. Nevertheless, the low-lying N-lone pair makes this end-on coordination mode the most energetically favorable. Here, the pyridine moieties act as moderate σ-donors and moderate π-acceptor ligands (Figure 1A), rendering them substitutionally labile under catalytic conditions. Even though this can be a desirable feature for some catalytic applications,^1^ a proportionally larger number of approaches focus on enhancing their kinetic stability against substitution. While this can be controlled by the nature of functional groups grafted on the pyridine ring, more commonly this is achieved through the incorporation as building blocks for meridionally (pincer) or facially (tripodal) coordinating ligands. As part of pincer systems,^2,3^ pyridine rings are common supporting (spectator) central fragments, and can be fine-tuned electronically, through the functionalization of the 4-position. Furthermore, functionalization in the 2,6-position allows a better control of the steric environment around the metal center. This approach has been used with a lot of success especially in constructing chiral catalysts, whose applications have been reviewed.^4^ The ease of functionalization of pyridine rings enabled them to serve as common fragments for the construction of tripodal ligands, with the tris(2-pyridylmethyl) amine scaffold (Figure 1B) being well-explored in synthetic models relevant for bioinorganic chemistry.^5^ Apart from their spectator role, N-bound pyridine fragments have been used to construct systems which are involved directly in bond-making and bond-breaking steps within substrate activation, i.e. they can function as active ligands. Here, the mechanistic aspects are often categorized and described under the larger concept of metal–ligand cooperativity (MLC). Pyridines are typically involved in two types of MLC: redox MLC and chemical MLC. Redox MLC typically relies on the ability of the supporting ligand to act as an electron reservoir and not on its structural involvement in bond-activation steps. As such pyridine fragments are known to be redox-active, reversibly storing one or two electrons on the heterocyclic core (Figure 1C).^6^ While the pyridine-based electron storage is more seldomly encountered in the ground state, metal-to-ligand charge transfer processes can be induced by photoexcitation, giving rise to a large palette of photocatalysts.^7^ Another important role is played by pyridines in constructing ligands which can operate via chemical MLC mechanisms (*i.e.*, where the ligand scaffold is structurally involved in bond activation steps). The reduced aromaticity of pyridine cores compared to *e.g*. arenes enables a more facile dearomatization, which can be triggered by the deprotonation of an acidic functional group installed in the α-position (*e.g.*, benzylic phosphine, benzylic sulfide, amine, alcohol, etc.).^8^ The reversibility of dearomatization constitutes the driving force for subsequent bond-activation steps, or for product release. While this activation mode often involves transition metals, p-block elements (*e.g*., boron) are also known to facilitate bond activation via pyridine aromatization-dearomatization cycles, relying on frustrated Lewis pairs (FLP) type mechanisms in the substrate activation steps.^9^

The end-on coordination mode is certainly the most encountered one; nevertheless, seldomly, pyridine ligands can adopt a side-on coordination mode, where the delocalized π-orbitals can donate into the metal d_π_-orbitals, in a binding mode similar to that of metal-arene complexes. This coordination mode is, nevertheless, less favorable due the reduced aromaticity of pyridines and their enhanced π-acidity compared to arenes. This coordination mode is more often encountered for early transition metals,^10^ and in cases where the N-lone pair is effectively shielded by sterically demanding substituents installed in the 2,6-positions.^11^ End-on coordination through one of the aromatic carbon atoms (*i.e.*, pyridinyl fragments) has been used to construct anionic pincer ligands, where the unligated pyridine N atom can be used for further functionalization.^12^

### Comparison between Pyridines, Diazines, and Triazines

**Figure 2:**
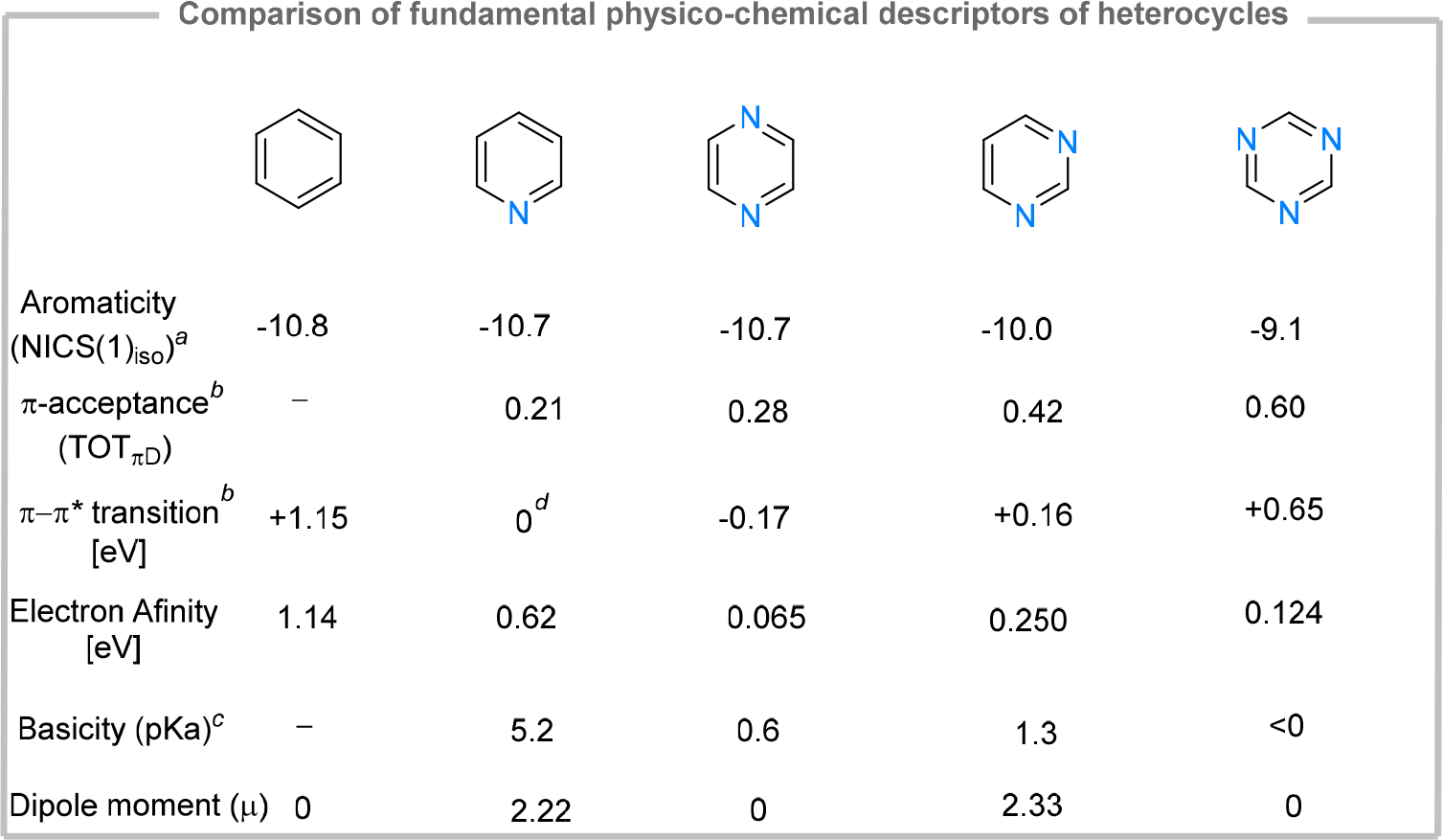
(a) NICS data; optimization: B3LYP/def2-TZVP/D3BJ, NMR: pcSseg-3. (b) Data from ref (16). (c) Data from ref (14). (d) Values are given relative to vertical excitation energy of pyridine: 4.94 eV.^16^

Given the very diverse chemistry of pyridine-based ligands, it can be construed as somehow surprising that the isosteric diazine and triazine analogues remain significantly less explored. The reasons behind this can be traced to the differences in electronic properties that result from introducing further nitrogen atoms in the heteroaromatic scaffold, and the more complicated synthetic routes that these heterocycles impose. The ability of the resulting motifs to function as a ligand is determined by the following descriptors, which will be discussed briefly (Figure 2): (1) aromaticity, (2) π-accepting properties, and (3) N-basicity.

While quantifying aromaticity is a challenging task and various scales are often defined,^13^ it is generally agreed upon that aromaticity in 6-memberd heterocyclic rings decreases with increase of N atoms. This can be rationalized in terms of a higher degree of electron localization on the nitrogen atoms which partially disrupts conjugation. The trend is reflected by various structural, computational and magnetic methods used to measure aromaticity in heterocycles.^14^ Using the nuclear independent chemical shift (NICS) method,^15^ a significant decrease in aromaticity can be inferred for pyrimidine and triazine, compared to pyridine and pyrazine (Figure 2). The lower degree of aromaticity of these heterocycles is reflected in their increased susceptibility to nucleophilic attack, which can become significant when considering ligand design (*vide infra*).

When describing the metal–ligand interactions of pyridines and, by extension, of diazines and triazines, the σ-donating and π-accepting properties need to be considered (Figure 1A). The overall π-accepting properties can be quantified by measuring the first vertical excitation energy by UV spectroscopy, which is commonly correlated to the π–π* energy gap. These measurements are often corroborated by computational methods (such as the total π-acceptance calculations, TOT_πD_). An increase in the number of nitrogen atoms corresponds to a stabilization of the LUMO (π*), therefore increasing the overall π-acidity (Figure 2). The effect is more important in pyrimidine (due to the asymmetry of charge distribution) and triazines (increased number of nitrogen atoms). This is corroborated by the values obtained through measuring the electron affinity (*i.e.*, the energy required for placing one electron in an unfilled orbital) of simple heterocycles via electron transmission microscopy.^17^ The other component of the metal–ligand bonding, the direct σ-donation ability is directly correlated with the stabilization of the lone pair, which can be inferred from Brønsted basicity. Measuring the p*K*_a_ values of the protonated bases reveals a sharp decrease in basicity with the number of nitrogen atoms (Figure 2). Compared to pyridine, the basicity of pyrazine and pyrimidine decreases by 4 orders of magnitude and becomes negligible for triazines.

### Considerations When Using Diazines and Triazines as Part of Ligand Scaffolds

A quick inspection of the difference in electronic properties of pyridine and its nitrogen-rich analogues hints at an interesting opportunity for ligand design, as all *N*-heterocycles herein considered are isostructural but have significantly different electronic and chemical properties. For metal-based catalytic applications, the variation of the heterocyclic core in ligand design offers the possibility of modulating the electronic properties, with minimal changes to the steric environment around the metal center. In principle, this decouples electronic effects from steric ones and allows the former to be studied independently, facilitating the extraction of important mechanistic information.

Nevertheless, the electronic properties of diazines and triazines require additional factors to be taken into consideration for ligand design, compared to the established pyridine analogues. An important consequence of reduced aromaticity is the increased susceptibility toward nucleophilic attack, for which, adequate steric shielding needs to be considered. This is especially relevant if the catalyst is envisioned to operate under strong nucleophilic conditions. Another important characteristic is the decreased Lewis-basicity of the N-lone pair in diazines and triazines, making them more susceptible to substitution compared to the pyridine analogues. This shortcoming can be circumvented by introducing strongly coordinating substituents adjacent to one of the heterocycle nitrogen atoms, enforcing a tridentate meridional coordination environment for the metal center (*e.g*., pincers). Lastly, nitrogen-rich building blocks are per definition polytopic with respect to metal coordination. Therefore, in order to control the coordination site, adequate kinetic shielding of the “free” nitrogen atom needs to be provided.

Nevertheless, despite possible challenges associated with ligand synthesis, employing diazines and triazines as building blocks for ligand design can offer opportunities unavailable to their pyridine congeners. New reactivity modes can be triggered by (1) the functionalization of the free nitrogen atom subsequent to complexation, (2) the increased π-acidity compared to pyridines, which can improve catalyst stabilities under reducing conditions, (3) the more energetically favorable population of ligand-based π*-orbitals, facilitating metal-to-ligand charge transfer, and (4) the reduced aromaticity of diazine and triazine cores can facilitate reversible ring dearomatization processes in chemical MLC.

This review therefore aims to showcase how these electronic characteristics of diazines and triazines can be capitalized upon in the construction of transition-metal based catalytic systems. Where available, the comparison with the analogous pyridine systems is also presented. While several diazine and triazine cores have been used to construct ligands relevant for the study of magnetic properties, photoluminescence or photocatalysts, these systems are beyond the scope of this review article. In some cases, systems for which catalytic properties have not yet been explored are presented if the ligand design bears strong similarities with catalytically active systems (*e.g*., present potential vacant sites for substrate binding).

## Modulation of Reactivity through Unligated N-Functionalization

### Diazines as Triggers for Remote-Site Functionalization

**Figure 3:**
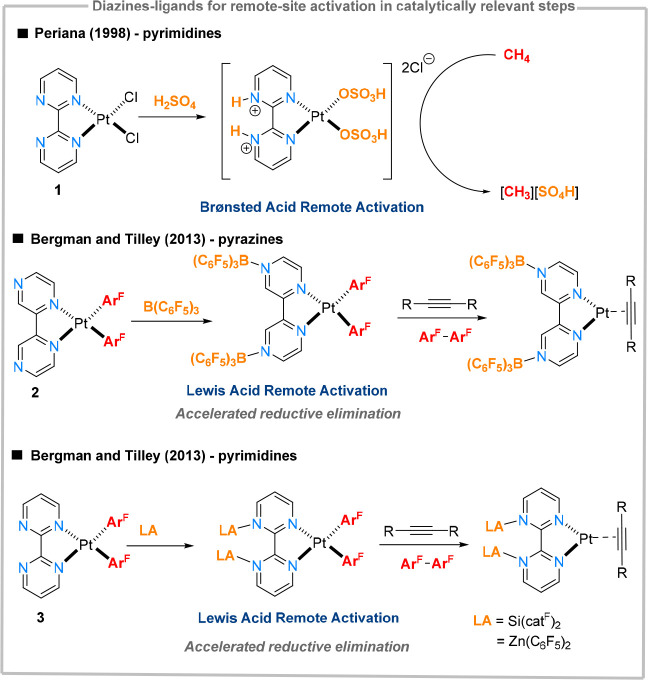
Remote-site functionalization effects in catalysis exemplified on diazine ligands.

Using pyrazine or pyrimidine ligands to enable electronic communication between two metal centers is a well-established approach to facilitate ligand-mediated metal-to-metal electron transfer. A famous example is represented by the Creutz-Taube complex. Comprising of two mixed valence Ru(NH_3_)_5_ units (Ru(II) and Ru(III)) bridged by a pyrazine ligand,^18^ this complex and its analogues played a fundamental role in understanding electron delocalization in mixed valence systems. Further exploration into this class of complexes lead to the development of redox active metal–organic frameworks and supramolecular coordination complexes, which found several applications in material chemistry^19^ and construction of photolumiescent materials.^20^ A prominent example of using diazines in catalytic applications represents the platinum-based system **1** (Figure 3), developed by Periana et al. (“Catalytica”), used for the catalytic methane conversion to methyl bisulfate.^21^ This system served as an inspiration for the development of other late-transition metal electrophilic C–H functionalization reactions.^22^ Here, the unligated pyrimidine nitrogen atom is protonated under the acidic reaction conditions, enhancing the π-acidity of the ancillary bipyrimidinium ligand. This effect increases significantly the electrophilicity of the platinum center, lowering the metal-based LUMO and therefore rendering it energetically favorable for accepting electrons of the σ-C–H from methane.^23^ This observation further paved the way for the design of proton and Lewis-acid responsive catalytic systems based on N-heterocyclic ligands. Pyrimidine and pyrazine fragments seem to be ideally suited for acting as ligand-cores, as they can bind a metal center through one nitrogen atom, while leaving the second nitrogen atom open for further functionalization. This allows the electronic tuning of a potentially catalytic system without a direct steric modification of the active site. Boron Lewis acids have, for instance, been shown to modulate the electronic properties of bis(pyrazine)platinum complexes (**2**, Figure 3), decreasing the reduction potential of the system by as much as 600 mV. This increased susceptibility toward reduction ultimately enhanced the rates of C–C reductive elimination by up to 8 orders of magnitude compared to cases where Lewis acids are not employed.^24^ A similar effect has been observed when using boron, silicon and zinc Lewis acids in combination with bis(pyrimidine)platinum complexes (**3**).^25^ Similar to Periana’s system, binding Lewis acids to diazines resulted in a lowering of metal-based LUMO, which can facilitate interactions with various σ- or π-basic substrates or can stabilize reduced metal states, thus accelerating reduction reactions.^26^ Similar catalytic effects have been observed in C–N coupling reactions of sulfonamides and pyridines.^24b,24c^ Binding protons or Lewis acids at the periphery of appropriately designed ancillary ligands in order to change electronic properties is a concept which was only relatively recently applied in molecular catalysis. However, the conceptually similar allosteric regulation of enzymes is a common mode of controlling catalytic activity in biological systems.^27^

### Repurposing Diazine-Based Ligands through Distal N-Functionalization

**Figure 4:**
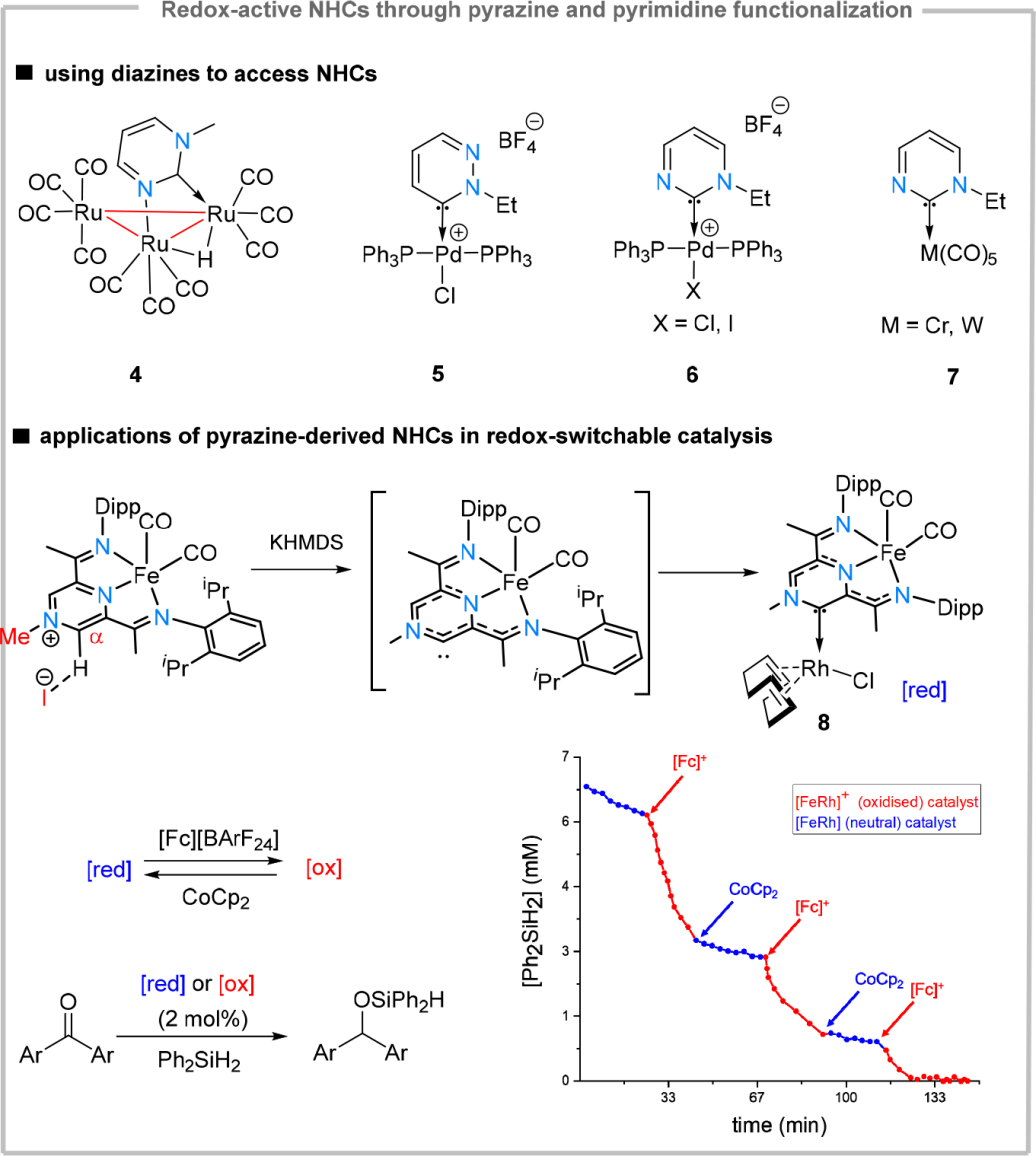
Generation of “abnormal” NHCs from pyrazines and pyrimidines and application in redox-switchable catalysis.

While distal functionalization of metal-diazine complexes can have a direct effect on the electronic properties of the metal center, it can also modify the reactivity of the ligand core, making it susceptible to new modes of activation. For example, alkylation decreases the p*K*_a_ of the adjacent C–H bond. Subsequent deprotonation in the presence of mild bases then provides an elegant entry into the chemistry of N-heterocyclic carbenes. While such approach is well established for other *N*-heterocycles (*e.g*., imidazoles^28^ and pyridines^29^), analogous approaches employing diazines as precursors have only been scarcely reported. Earlier examples involving pyrimidine-derived NHC metal-complexes were prepared via cycloaddition reactions of Fischer chromium and tungsten carbenes with imines.^30^ Nevertheless, it has soon emerged that direct routes, relying on N-alkylation, followed by deprotonation and metalation can offer a more expedient entry into this class of complexes. For example, Cabeza and co-workers used alkylated pyrazinium and pyrimidinium precursors to access multimetallic ruthenium NHC complexes (*e.g*., **4**, Figure 4), where the carbene center acts as a bridge between two metal centers.^31^ Examples where the NHC lone pair is donated into a single metal orbital were obtained for palladium, tungsten and chromium complexes (*e.g*., **5**–**7**) using alkylated pyridazine- and pyrimidine-derived precursors.^32^ In contrast to the pyridine-derived analogues, no catalytic transformations have been reported for **4**–**7**. Nevertheless, the interest in this class of complexes was fueled by their interesting electronic structure and by the mesoionic nature of the carbene derivatives, which has been also explored via computational studies.^33^ A first catalytic application of diazine-derived NHCs was enabled by using an iron-based pyrazine-diimine (P^Pz^DI) redox-active ligand precursor. Methylation of the distal nitrogen atom, followed by deprotonation and metalation with [Rh(COD)Cl]_2_ furnished facile access to **8**, which is a heterobimetallic ditopic NHC, binding two redox-active metals through a redox-active ligand.^34^ Complex **8** could be easily oxidized in a reversible fashion at very mild peak potentials (*E*_1/2_ = −600 mV). Interestingly, the oxidation is ligand-based, therefore conserving the Rh(I) oxidation state. This, in turn, allowed the rhodium center to engage in redox reactivity, which could be utilized for the hydrosilylation of olefins and ketones. While both oxidized and reduced forms were catalytically active, the oxidized form showed 1 order of magnitude enhancement of the reaction rate. *In situ* oxidation and reduction in the presence of ferrocenium salts and cobaltocene respectively allowed multiple switching between the two kinetic regimes. Therefore, the (P^Pz^DI)Fe(CO)_2_-derived NHC acts as a redox switch, which is able to operate at significantly more milder potentials compared to the well-established metallocene-based redox switches.^35^

## The Effect of Increased π-Acidity in Catalytic Transformations: The Case of Redox-Active Ligands

**Figure 5:**
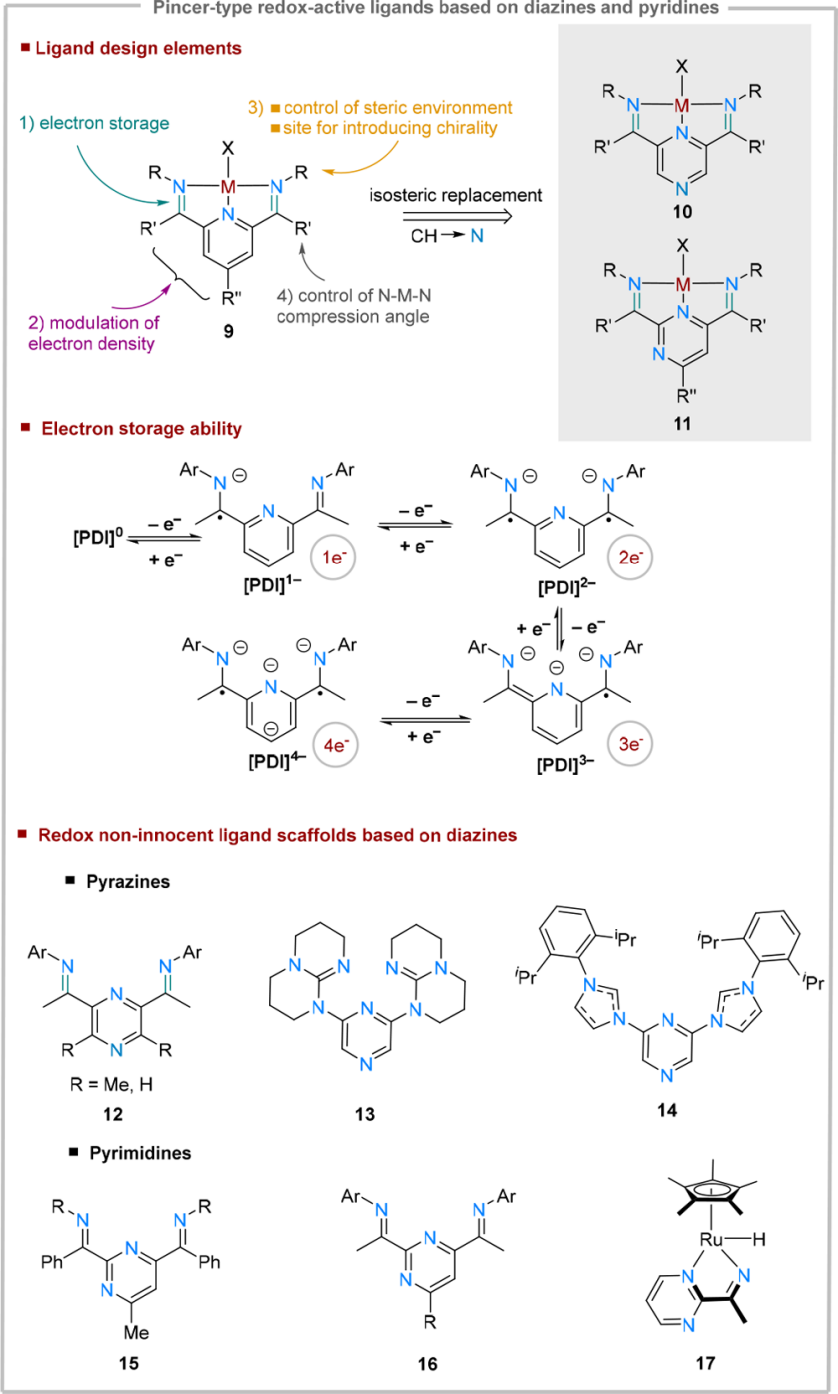
Ligand-design elements for pyridine-diimines (PDI), their diazine-analogues, and other examples of diazine-based redox-active pincers.

A class of pincer ligands where pyridine fragments are frequently employed are redox-active ligands. While the pyridine core can be itself redox active (Figure 1C) under strong reducing conditions, it is often the functional groups grafted on the ring that function as an electron reservoirs. Ligands based on pyridine-imines and pyridine-diimines (PDIs), (**9**, Figure 5) have emerged as highly versatile redox-active platforms, capable of binding metals across all blocks of the periodic table.^36,37^ In catalysis, these ligands have gained significant attention from the organometallic community in 1998, when Brookhart and Gibson reported simultaneously that the activation of (PDI)FeCl_2_ and (PDI)CoCl_2_ complexes with methylaluminoxanes (MAO) generated highly active species in olefin polymerization.^38^ Apart from their high activity, these systems did not promote chain walking and therefore enable the synthesis of high density polyethylene. Through depth mechanistic investigations of these catalytic applications^39^ the electronic complex structures of these complexes surfaced. It was soon recognized by Wieghardt,^40^ Budzelaar,^41^ and Chirik^42,43^ that ligand-based redox processes play an important role in the *modus operandi* of these ligand systems. This implies that these systems are capable of storing reversibly up to four electrons under very strong reducing conditions: two electrons at the imine arms and, subsequently, two more electrons at the pyridine core (Figure 5).^44^

In an effort to modulate the catalytic activity in olefin polymerization, while not altering the steric bulk at the metal center and preserving the ligand-based redox processes, isosteric replacements at the heterocyclic core were explored. As increasing the nitrogen content of the heterocycle is correlated with milder reduction potentials (see Introduction), it is expected that the resulting diazine-base ligands would facilitate more metal-to-ligand charge transfer processes. An initial report, published in 2006, described the activity of iron-based pyrazine-diimine (P^Pz^DI) complexes (type **10**, Figure 5) in ethylene polymerization catalysis, where the corresponding complexes performed less efficiently compared to the pyridine analogues.^45^ Here, the pincer ligand was constructed via a Minisci coupling, relying on the 2,6-acylation of pyridine in the presence of pyruvic acid. Nevertheless, later reports supported by crystallographic evidence, demonstrated that this synthetic route in fact yields selectively the undesired 2,5-isomer.^26a,46^ The pyrazine-diimine system with the pincer (2,6) connectivity (**10**) was later synthesized via an alternative reaction pathway, but the catalytic activity of the related iron complexes in olefin polymerization was not reinvestigated. Further reports expanded on this ligand class by introducing methyl groups on the pyrazine core which offer better steric shielding of the distal nitrogen ligand (**12**).^26b,26c^ Other redox-active ligands based on pyrazine cores use guanidines (**13**)^47^ and NHCs (**14**)^48^ as redox-active fragments, and have been studied due to their photoemissive properties.^49^

Other than the NHCs derived from pyrazine-diimines described above (Figure 5), applications of pyrazine-diimine ligands in catalysis remain unreported. In contrast, pyrimidine-diimine (P^Pym^DI, type **11**) analogues have been studied more intensely. An early report on the use of pyrimidine-based systems (**15**) in catalysis was published in 2003, by exploring iron and cobalt based pyrimidine analogues of the well-known pyridine-diimine systems (PDI) (type **9**, Figure 5).^36c^ For synthetic accessibility, phenyl rings were introduced in the α-position to the N=C functionality. However, the enhancement of π-acidity imparted by the combined effects of the pyrimidine ring and α-phenyl groups destabilized the metal–ligand interactions (*vide infra*, Figure 9). These effects are also mirrored in the catalytic activity for olefin polymerization: the reaction rates of iron complexes based on P^Pym^DIs are comparable to the ones of PDIs in the first reaction half-life but rapidly plummet subsequently. An improvement of the stability of these systems was reported in 2021, when a better balance between ligand π-acidity and stability of metal–ligand bonds was achieved through the design of **16**.^50^ This led to more robust catalytic systems for metal-mediated cycloaddition and hydroelementation transformations, which will be discussed in more detail in the next section. Ligand-based redox processes of pyrimidine-imine fragments were recently investigated in the context of ruthenium hydride complexes (**17**) for the catalytic hydrogenation of various N=N and M=N bonds.^51^ Here, it was demonstrated that that binding a pyrimidine-imine redox-active ligand to a Cp*RuH fragment leads to a significant lowering of the Ru–H bond-dissociation free energy (BDFE), facilitating hydrogen atom transfer (HAT) reactions.

### Comparison of Electronic Structure and Catalytic Activity of Pyrimidine-Diimine and Pyridine-Diimine Iron and Cobalt Complexes: Structure–Activity Relationships

**Figure 6:**
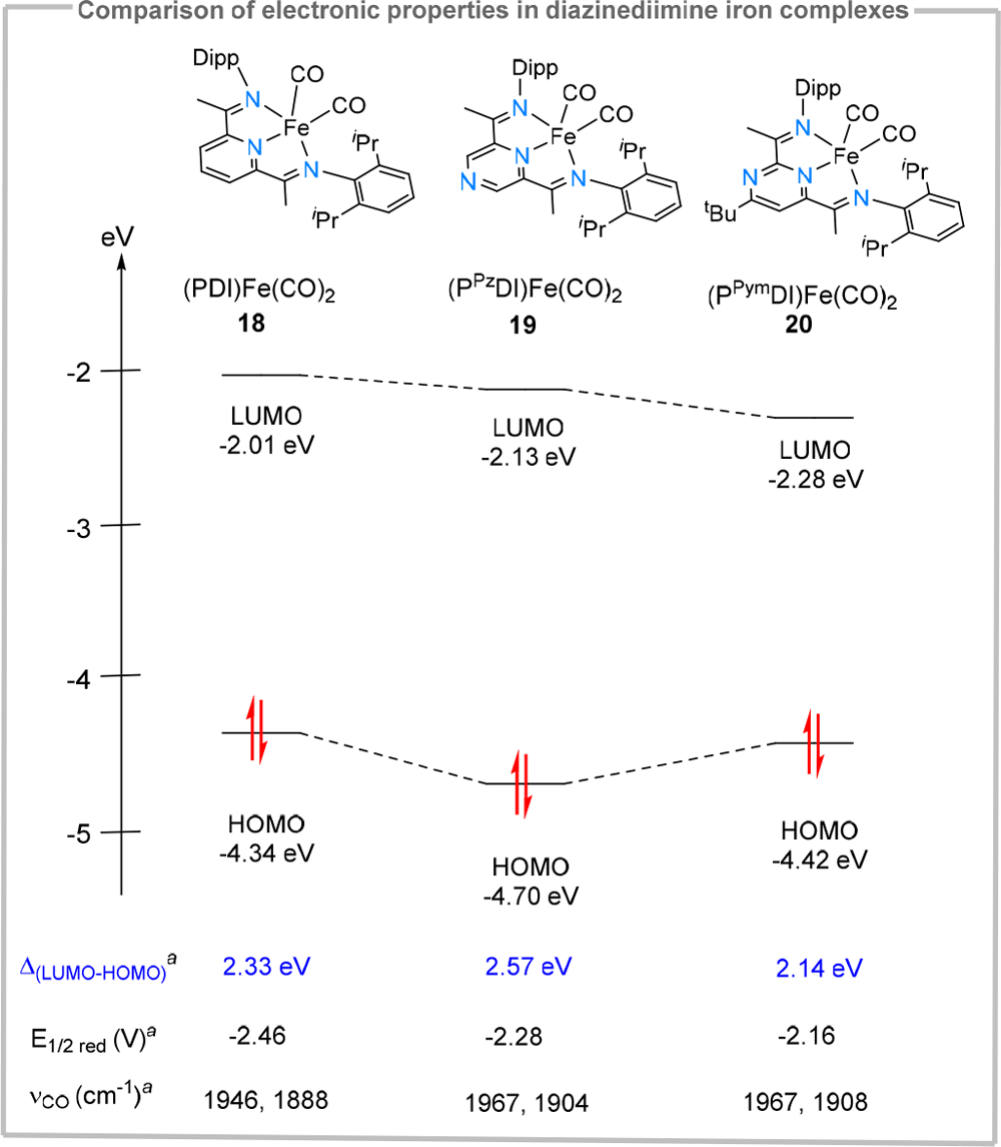
Calculated frontier orbital energies in (PDI)Fe(CO)_2_ and their diazine analogues. Comparison of experimental redox-potentials and ν_CO_ stretching frequencies from IR spectroscopy. ^*a*^Data taken from ref (53).

Despite their suitability as isosteric replacements for pyridine fragments, there are only a few studies where electronic differences between various diazines and pyridines have been systematically inverstigated. An early computational study already enunciated key differences in electronic properties,^52^ but it was only in 2022 when these differences were investigated experimentally.^53^ Iron–carbonyl complexes were initially used as models due to their stability across multiple redox states. Complexes isostructural to (PDI)Fe(CO)_2_ (**18**, Figure 6)^54^ were synthesized, where the supporting heterocycle was formally replaced with pyrazine (**19**) and pyrimidine (**20**). The electronic structure was then compared using a combined spectroscopical and computational approach. Essentially, formal CH → N replacements at the supporting heterocycle did not change the nature of ligand-based redox processes, and therefore diazine-diimine based systems (P^Pz^DI)Fe(CO)_2_**19** and (P^Pym^DI)Fe(CO)_2_**20** are expected to show similar electron storage properties as the pyridine analogue (see Figure 6).

**Figure 7:**
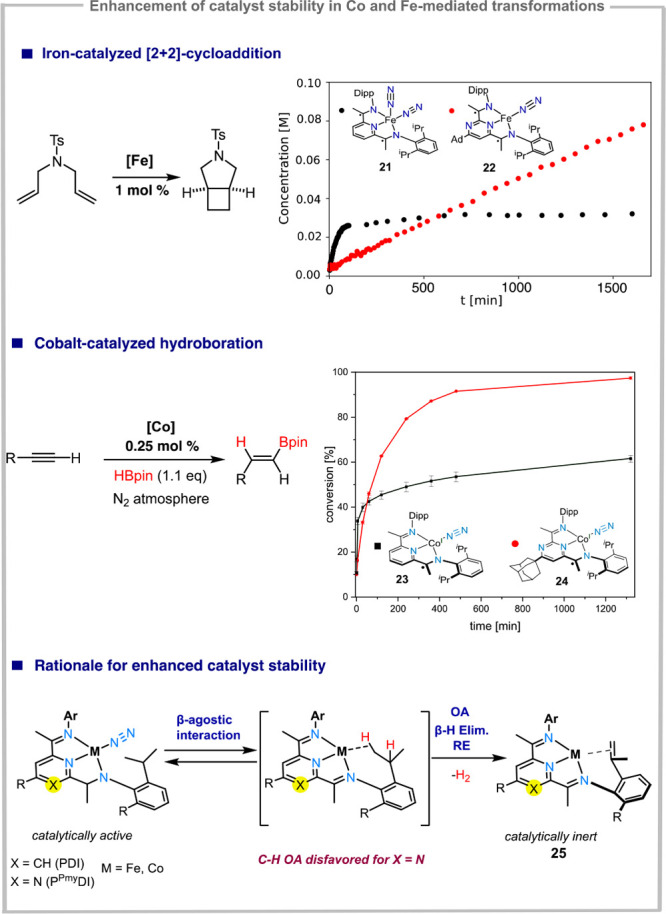
Pyrmidinediimine (P^Pym^DI) iron and cobalt complexes and examples of enhanced stability compared to the pyridine analogues under [2 + 2]-cycloaddition and hydroboration conditions.

Nevertheless, due to the different electronic properties of the supporting heterocycle, significant differences were observed in the redox potentials and π-acidity, as reflected by CV measurements and IR-stretching frequencies (Figure 6). DFT studies further corroborated the spectroscopic findings and concluded that, while the electronic structure is invariant for the series explored, a stabilization of the LUMO and a narrowing of the HOMO–LUMO gap was observed for the (P^Pym^DI)Fe(CO)_2_ complex (**20**). Similar conclusions could be drawn also for the more catalytically relevant (P^Pym^DI)Fe(N_2_) **22** (Figure 7).

The subtle differences in electronic structure were also reflected in the catalytic activity of P^Pym^DI-based iron and cobalt complexes compared to their pyridine analogues. Two major classes of reactions were addressed: iron-catalyzed cycloaddition reactions, and cobalt-catalyzed hydroboration. For iron-catalyzed [2 + 2]-cycloaddition reactions (Figure 7, top), iron-bound (P^Pym^DI)-ligands proved to be significantly more stable than the isosteric pyridine-based analogues: under the same reaction conditions, PDI-based platforms showed deactivation during the first reaction half-life, while pyrimidine-based systems were stable throughout the reaction, as indicated by the measured kinetic profiles.^55^ The same behavior was observed in the case of cobalt-catalyzed hydroboration of olefins and alkynes, using (P^Pym^DI)Co(N_2_) **24** as precatalysts.^56^ Compared to the pyridine-analogue (**23**), the pyrimidine isostere shows a reduced reaction rate, which reflects the more electron deficient nature of the catalytic system, increasing the activation barrier for the turnover limiting step (oxidative addition). In the [2 + 2] cycloaddition reaction, this decrease in rate could be circumvented by fine-tuning the flanking N-Aryl groups. Using bulkier flanking groups lead to significant increases in reaction rates, which allowed the synthesis of a broad range of cyclobutane-fused pyrrolidines, piperidines and azepans.^55^ For both cobalt and iron complexes, high-catalytic efficiency was attributed to the π-acidic heterocyclic ring, which disfavors catalyst deactivation pathways. A common catalyst deactivation pathway determined by the kinetically shielding Dipp (Dipp = diisopropylphenyl) and Mes (Mes = mesytilene) groups in the coordination sphere of reduced metal centers is associated with the high tendency of these groups toward cyclometalation, subsequent to C–H oxidative addition. If a syn-coplanar conformation can be reached, β-hydride elimination, followed by reductive elimination of H_2_ yields an intramolecular metal-olefin complex (**25**) which is catalytically inactive (Figure 7, bottom). In the context of (PDI)Fe complexes for example, such reaction side-products have been isolated by Chirik et al.^57^ Since the π-acidic pyrimidine ring destabilizes metal-(C–H) backbonding interactions, this deactivation pathway is rendered more unfavorable for the (P^Pym^DI) complexes compared to their pyridine analogues.^55^

**Figure 8:**
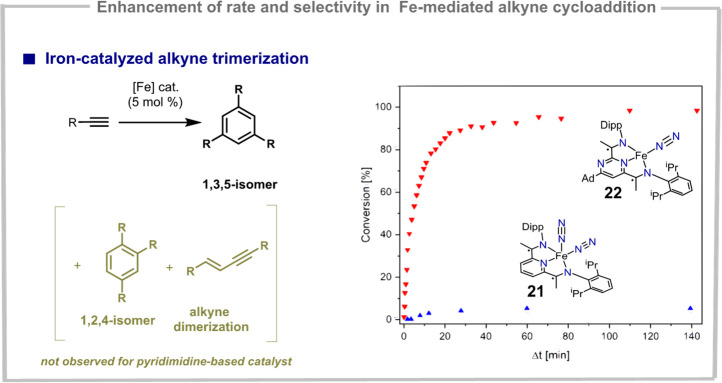
1,3,5-Selective trimerization of alkynes catalyzed by (P^Pym^DI)Fe-systems.

Replacing a pyridine ring with pyrimidine can also trigger significant changes in product selectivity. This phenomenon was observed in the context of iron-catalyzed alkyne trimerization. Namely, when using alkyl-substituted terminal alkynes as substrates, (P^Pym^DI)Fe-systems were able to promote a [2 + 2+2]-cycloaddition reaction which proceeded with a rare 1,3,5-regioselectivity.^50^ In the case of the pyridine analogue, the reaction was sluggish and unselective. Specifically, under the same reaction conditions, mixtures of various arene regioisomers and alkyne dimerization products were observed (Figure 8). While the determining factors of regioselectivity for (P^Pym^DI)Fe systems remain unknown, the isolation of a 1,3-substituted ferracycle suggests that the reaction mechanism relies on a coordination-cyclization-insertion-reductive elimination reaction sequence, where the cyclization and insertion steps proceeded with high regioselectivity. It is interesting to note that this selectivity is not influenced by the steric environment around the metal center (controlled by the Dipp flanking groups) but by the electronic properties of the supporting pyrimidine ring.

### Limitations

**Figure 9:**
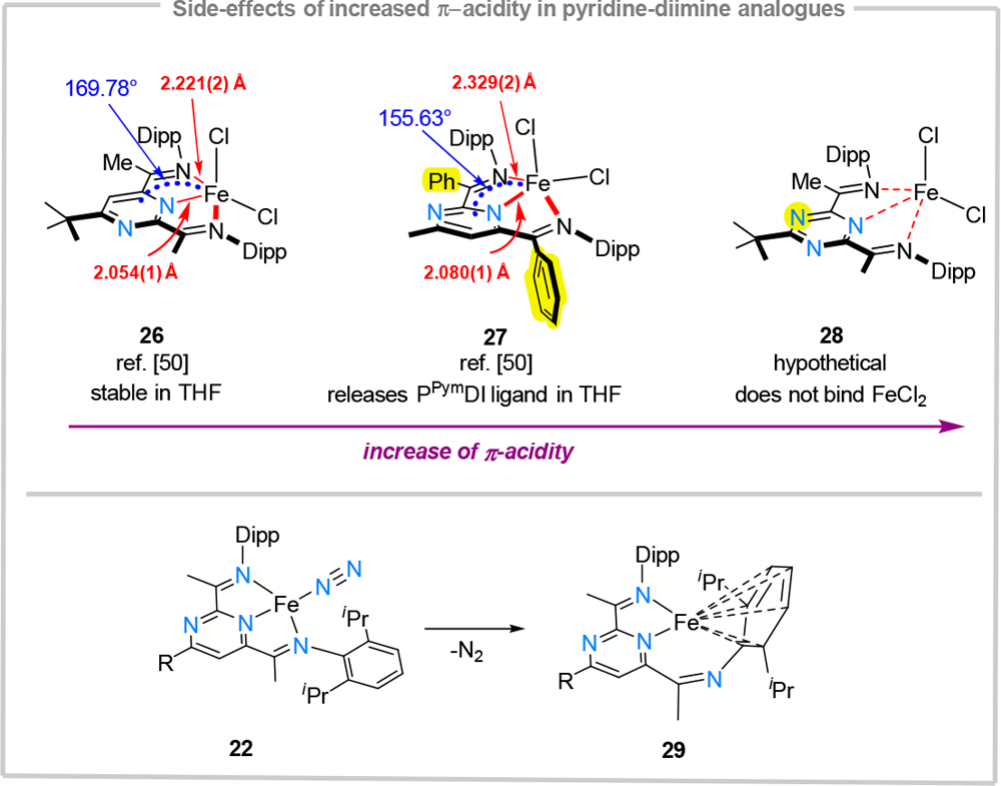
Observed deactivation pathways and limitations of (P^Pym^DI)Fe-complexes.

The examples presented above demonstrate potential advantages of considering CH–N isosteric replacements on heterocyclic rings when catalytic transformations are considered. Such replacements can be beneficial especially when catalyst stability issues need to be addressed, or when the nature of the turnover limiting step mandates a more electron deficient catalyst (*e.g*. for reductive elimination). Nevertheless, when considering a chelating ligand design relying solely on M–N interactions, a fine balance between ligand electron deficiency and stability of metal–ligand interactions needs to be struck. For example, introducing π-accepting phenyl groups adjacent to imine fragments, or further making CH-N isosteric substitutions on the heterocyclic core (*e.g*., using triazines) causes a strong increase in π-acidity. This determines a stronger localization of the N-lone pairs, resulting in an increase in substitutional lability of the chelate ligand. In more extreme cases, metal binding is disfavored altogether. For example, the methyl-substituted **26** is stable in THF solutions and the iron center is almost coplanar with the pyrimidine-based chelate (Figure 9). In contrast, introducing a phenyl group (**27**) reduces the kinetic stability of the complex, which undergoes facile ligand substitution reactions in coordinating solvents (*e.g*., THF).^50^ Moreover, crystallographic evidence shows that compared to **26**, the iron center is no longer coplanar to the pyrimidine-based chelate and the Fe–N interactions are significantly elongated. Compared to pyrimidine-based **26** and **27**, the triazine-based **28** does not bind FeCl_2_ and therefore metal centers with higher Lewis-acidity or in lower oxidation states need to be considered for this class of ligands. Another side-reaction that needs to be taken into consideration for π-acidic M-N_2_ complexes is their reduced stability under vacuum, as a result of destabilization of M-N_2_ bonds. For iron complexes, this can lead to the formation of Fe(η^6^-Dipp) complexes (i.e., **29**), which blocks potential coordination sites for the incoming substrates (Figure 9).^50^ Similar processes have also been observed for electron-deficient pyridine-diimine systems.^58^

## Chemical Metal–Ligand Cooperativity in Diazines and Triazines for Catalytic Transformations

**Figure 10:**
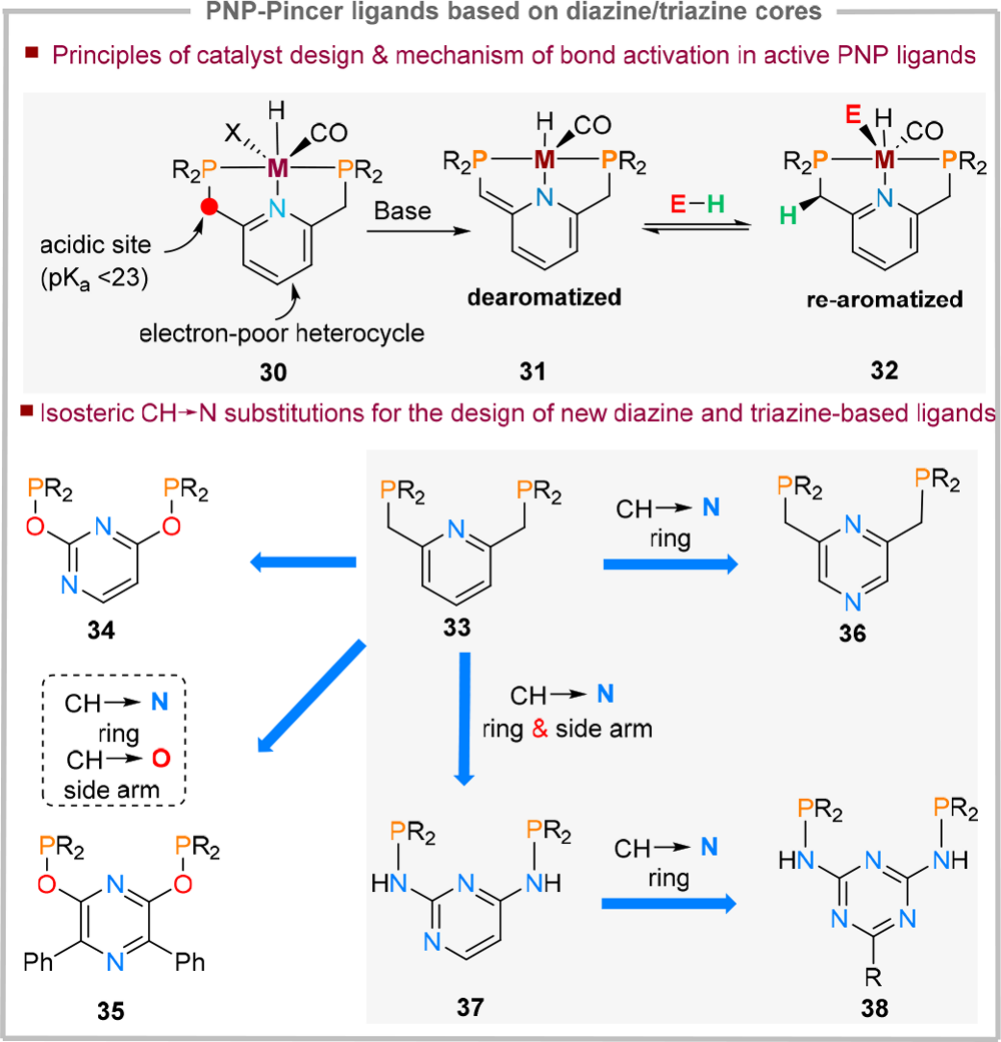
Chemical MLC mechanisms of pyridine-based PNP-ligands and examples of known diazine and triazine analogues.

Since the π-acidic nature of nitrogen-rich heterocycles can reduce metal–ligand bond stability, a vast number of approaches combine diazine and triazine cores with strong σ-donating ligands. A particularly attractive approach is to use neutral PNP-type ligands (**30**, Figure 10) as blueprints and formally perform ring CH → N isosteric substitutions, yielding **34**–**38**. PNP scaffolds based on pyridine-cores functionalized with benzylic phosphines are well-explored in the context of chemical metal–ligand cooperativity, where base-mediated deprotonation of the acidic CH_2_ group triggers pyridine dearomatization (**31**), which is regained upon reaction with an incoming substrate (**32**).^8^ Nevertheless, depending on the precatalyst activation mode, these ligands can also function as classical (spectator) ancillary ligands.

Analogously, for the resulting diazine and triazine ligands, two classes can therefore be envisaged: *(i)* spectator ligands–ligands which can modulate metal electronic properties and Lewis acidity but do not participate directly in bond making and bond-breaking steps and *(ii)* active ligands–where the ligand backbone suffers a (transient) chemical modification in one catalytic step or more.

### Spectator PNP-Ligands Based on Diazine Cores

**Figure 11:**
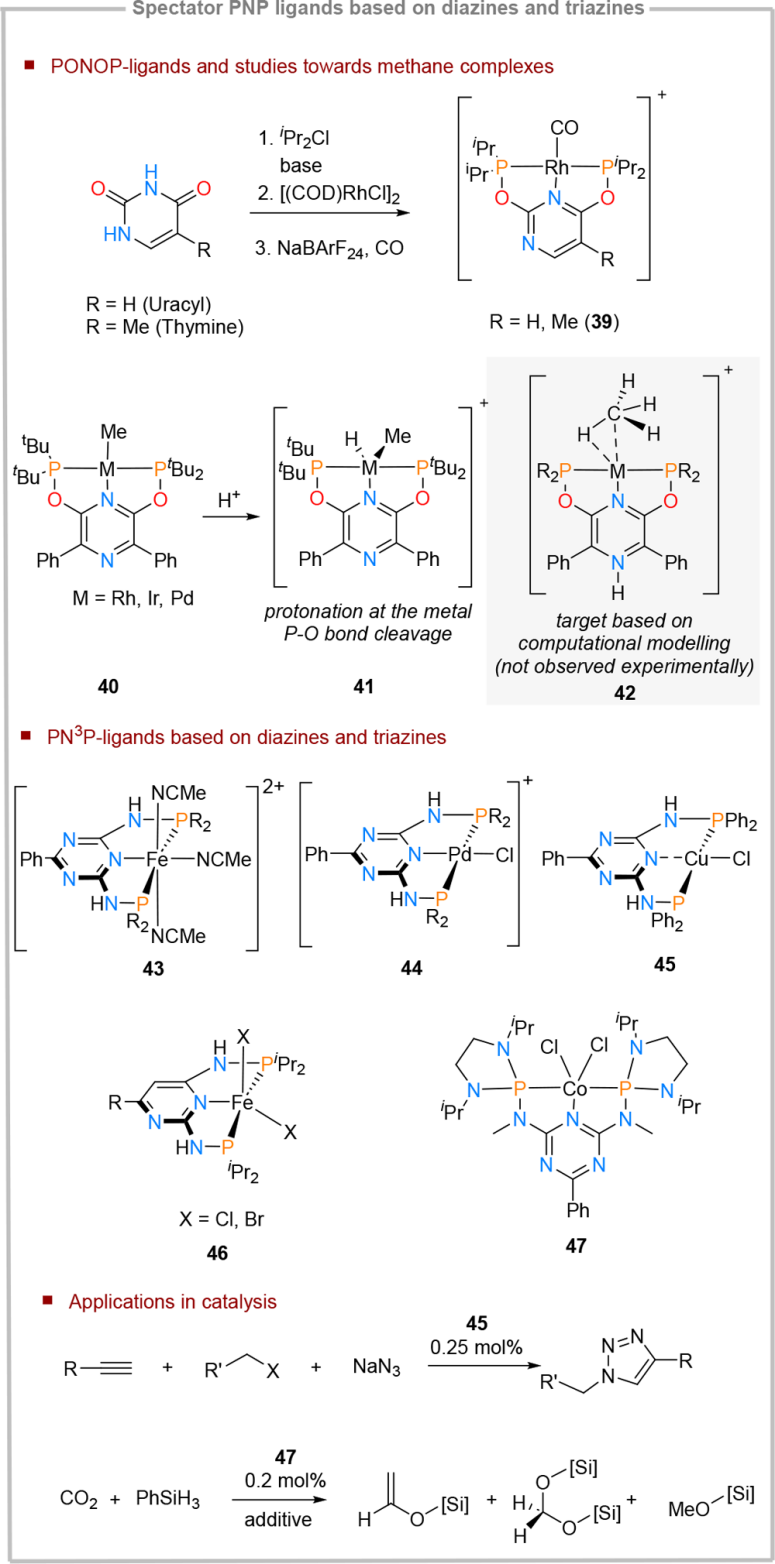
Diazine- and triazine-based PNP ligands as actor ligands.

Spectator ligands derived from PNP-scaffolds type **39** (Figure 11) can be derived from pyrimidine-based nucleic bases (*e.g.*, uracil and thiamine). In the presence of phosphines, the target ligand scaffold containing O–P linkage is in equilibrium with the N–P linkage form,^59^ while after complexation, only the form **39** (Figure 11) is obtained.^60^ The synthesis of the corresponding carbonyl complexes confirmed that **39** is significantly more electron deficient than the isostructural pyridine-analogue, as determined by IR spectroscopy. No further reactivity or catalytic studies were reported for this system.

The analogous structural motif (**40**, Figure 11) based on a pyrazine core was explored in an attempt to synthesize stable complexes containing metal–methane interactions.^61^ Multiple metal centers (Rh, Ir, and Pd) were investigated. The pursuit was motivated by computational studies which revealed that ligands bearing a protonated pyrazine core type **42** can enhance methane binding by ca. 5–7 kcal/mol compared to the pyridine analogues. This stabilization is a result of pyrazine protonation; unprotonated pyrazine cores and their pyridine analogues show similar methane binding affinities. Nevertheless, this hypothesis could not be verified experimentally, since pyrazine protonation at the distal nitrogen atom could not be achieved: under acidic conditions, protonation either occurs at the metal center (**41**) or triggers P–O bond cleavage.

Triazine- and pyrimidine-based PNP complexes bearing azine-phosphorus NH linkages were also investigated. This ligand design was initially explored in order to study the possibility of building intermolecular or ion pair hydrogen bonding interactions facilitated by the acidic N–H functionalities. For the triazine-bearing cores, the first examples were published in 2006,^62^ disclosing the synthesis of iron and palladium complexes (**43** and **44**), using a range of substitution patterns at the phosphorus atom. Recently, the copper analogues were also reported (**45**). Nevertheless, unlike **43** and **44**, the copper complex **45** is substitutionally labile, being prone to the formation of aggregates, triggered by either triazine or phosphine dissociation. This notwithstanding, **45** is highly efficient in mediating azine-alkyne click reactions, requiring only low catalyst loadings.^63^ The analogous pyrimidine-ligands have been disclosed three years later,^64^ and were explored in the context of studying differences between CO binding at iron centers (**46**) in solution and in solid state. In all cases, the reactivity of the diazine and triazine analogues mirrors closely the one observed for the pyridine analogues. Recently, a triazine-based PN^3^P ligand which bears NMe linkages between the core and the phosphine groups (**47**) was disclosed.^65^ The phosphines are functionalized with chelating amines fragments, significantly enhancing the σ-donation ability. This is complemented by the strong π-accepting properties of the triazine core. The cobalt complexes of this ligand were explored in the context of catalytic CO_2_ silylation, revealing remarkable activity. Interestingly, the product distribution resulting from the silylation reaction (*i.e.*, the formation of silyl formate, bis(silyl)acetal, or methoxysilane) could be efficiently controlled by choosing the appropriate precatalyst activation conditions. Moreover, computational studies revealed that the triazine core plays an important role in modulating the hydricity of the Co–H species generated under the catalytic conditions, which subsequently affects the energy span of the CO_2_ insertion step.^65c^

### Active PNP-Ligands Based on Pyrazine Cores

**Figure 12:**
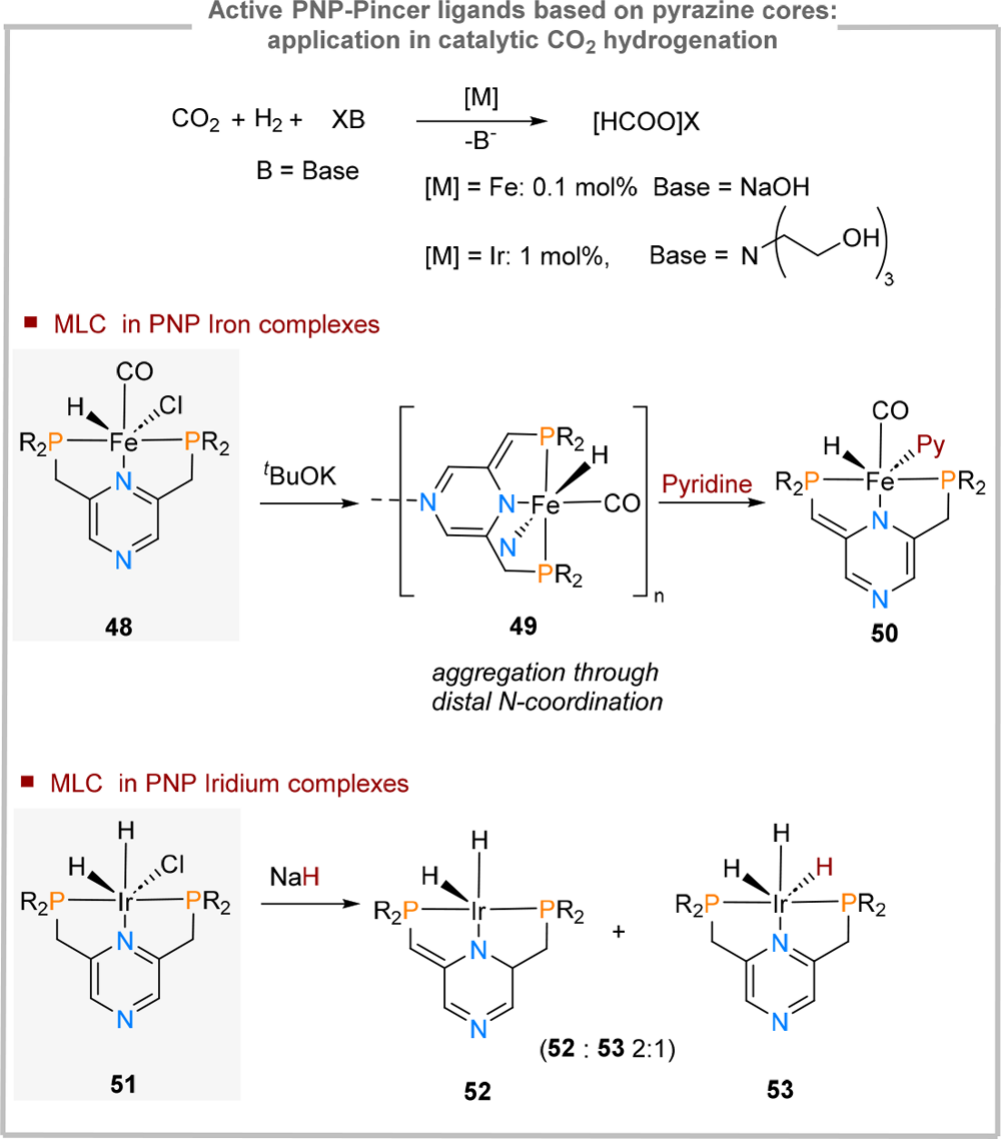
Pyrazine-based PNP active ligands in CO_2_ hydrogenation catalysis.

In the examples presented above (**43**–**47**) the diazine and triazine-based ligands do not suffer chemical modifications during the various reactivity studies and are therefore “spectators” in this respect. An opportunity to combine chemical metal–ligand cooperativity via core aromatization/dearomatization sequences (Figure 10) with remote-site functionalization (Figure 3) is the design of complexes type **48**.^66^ This ligand scaffold relies on a pyrazine core bearing two benzylic phosphine functionalities. The study of base-triggered core dearomatization was performed in the context of iron-mediated CO_2_ hydrogenation. While the resulting complex **49** could bind CO_2_ in a cooperative fashion (Figure 12), it also underwent aggregation through the participation of the distal pyrazine nitrogen atom, which engages in intermolecular Fe–N coordination and therefore reducing the solubility in common solvents (*e.g*. THF). Discrete (PNP)Fe units could be reobtained in the presence of strong donors (e.g., pyridine, **50**). Complex **48** was found to catalyze hydrogenation of CO_2_ in good turnovers at very low catalyst loadings.

A subsequent report on CO_2_ hydrogenation used the same ligand system but explored the reactivity of iridium-based catalysts.^67^ While the catalytic runs were performed using triethanolamine as a base, for the stoichiometric studies, sodium hydride was employed in the stoichiometric studies. Core dearomatization was inferred from NMR studies (**52**), but it was observed that NaH can also act as a nucleophile, undergoing a salt metathesis reaction with the starting material **51**. Interestingly, no aggregation similar to **49** and no nucleophilic attack on the pyrazine core were reported. In catalytic CO_2_ hydrogenation, the pyrazine-based **51** showed slightly higher turnover numbers compared to the pyridine analogue (98000 vs 88000).

### PNP-Ligands Based on Triazine Cores in Catalysis

**Figure 13:**
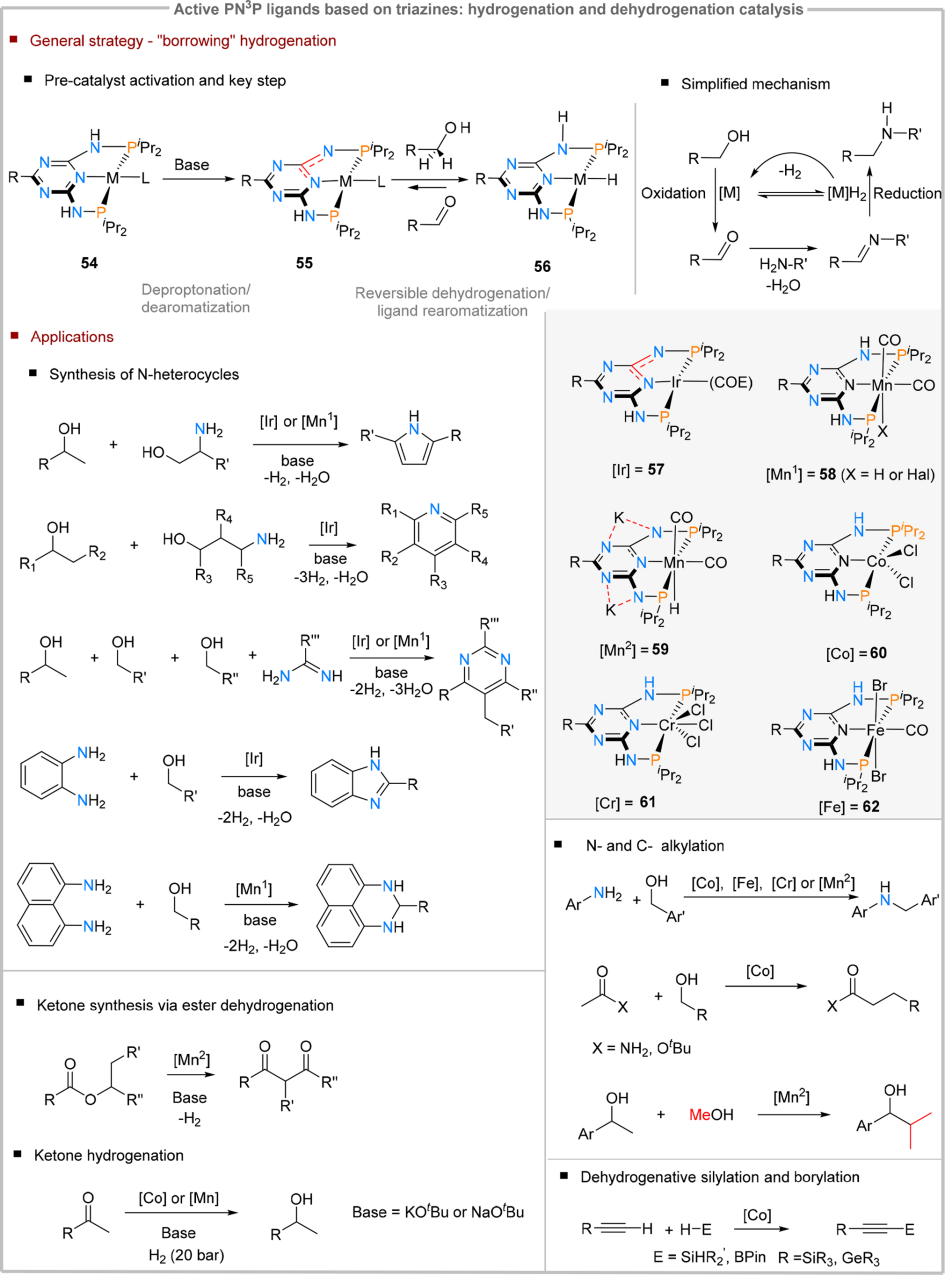
Triazine-based PNP complexes in MLC-type bond activation reactions and their applications in catalysis.

Triazine cores have been extensively used for the design of PN^3^P pincer systems and combine the following elements which make them attractive for use in chemical metal–ligand cooperativity (MLC): *(i)* a highly π-deficient heterocyclic core with reduced aromaticity compared to pyridine cores, *(ii)* strong σ-donating phosphines which stabilize metal–ligand interactions, compensate for the withdrawing effect exerted by the triazine fragment and enforce a κ^3^ -coordination mode (*e.g*., pincer), and last *(iii)* an acidic NH functionality which can trigger core dearomatization via deprotonation. The first report of this ligand motif was in 2006 by Kirchner et al. in the context of coordination chemistry studies on iron and palladium complexes.^62^ Nevertheless, their ability of performing MLC-type reactivity in catalytic transformations was only demonstrated by Kempe et al. in 2013, when iridium-based PN^3^P-complexes (**57**) were used for pyrrole synthesis through dehydrogenative and dehydrative condensation of alcohols and amines (Figure 13).^68^ The dehydrogenation mechanism mirrors the one established for pyridine-based PNP-pincers using CH_2_ and NH linkers between the phosphine and the aromatic core:^8,69^ The acidic N–H functionality in the precatalyst (**54**) is deprotonated in the presence of a base, yielding a highly reactive imine bearing a dearomatized triazine (**55**).

This species engages in cooperative dehydrogenation of an alcohol substrate, generating a metal hydride and an aldehyde. The resulting C=O functionality then reacts with an amine through dehydrative condensation, and the resulting imine is reduced by the metal-hydride. This acceptorless (de)hydrogenation methodology is applicable for a wide range of transition metals in several organic transformations, and distinguishes itself by the mild reaction conditions and the benign reaction byproducts (usually hydrogen and water).^70^ A summary on the catalytic transformations based on this concept using triazine-bases PNP active ligands is outlined below (Figure 13).

### Synthesis of N-Heterocycles

Acceptorless dehydrogenation has been employed in the synthesis of several types of heterocycles. This synthetic strategy relies on using alcohols and aminoalcohols as coupling partners which, upon condensation, yield an aromatic heterocycle resistant to hydrogenation. Both iridium- (**57**) and manganese-based (**58**) systems^71^ are highly active, often only requiring catalyst loadings under 0.5 mol %. One of the earlier applications of triazine-based PN^3^P in heterocycle construction was the synthesis of pyrroles.^72^ Through this method, a large variety of pyrroles with different substitution patterns could be synthesized. Interestingly, the manganese catalyst showed higher activity than the iridium variant, while the iron and cobalt analogues remain inactive for this transformation.^72b^

The same type of methodology using appropriate substrates could also be extended to the synthesis of 6-ring aromatic heterocycles (*i.e.*, pyridines and pyrimidines).^73^ A wide variety of regiosubstituted pyridines could be obtained using the iridium-based precatalyst **57** in the presence of a base.^73a,73b^ In a similar fashion, pyrimidines can also be accessed using iridium and manganese-based triazine systems.^73c,73d^ Notably, the manganese-triazine complex (**58**) shows substantially higher activity compared to the pyridine-analogue.^73d^ Using anilines as substrates also allows an efficient entry into the synthesis of benzimidazoles and quinoxalines, by using either alcohols or 1,2-diols substrates in the presence of iridium-based **57**.^74^ This could be extended to 1,8-diaminonaphtalene-substrates, which, upon dehydrogenative condensation with benzylic alcohols, yields 2,3-dihydro-1*H*-perimidines in the presence of manganese catalyst (**58**).^75^

### Amine and C-Alkylation

**Figure 14:**
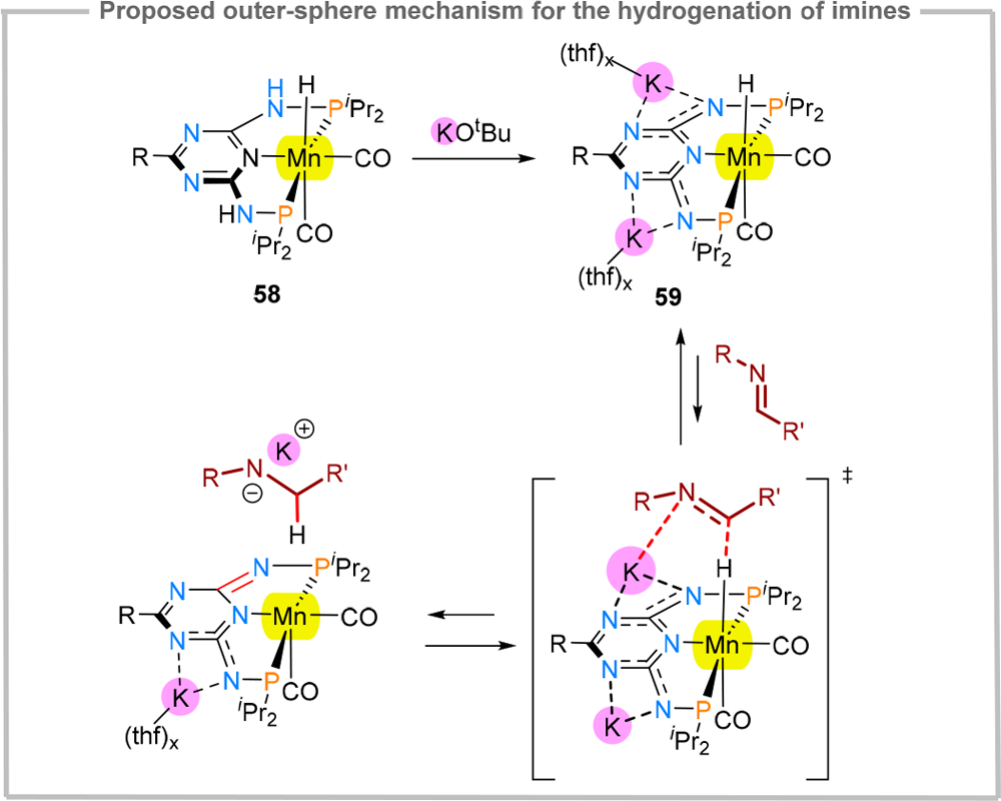
Key steps in imine reduction, highlighting the role of secondary triazine···K···substrate interactions.

The heterocycle synthesis described above can be regarded as an interrupted N-alkylation following the mechanism described in Figure 13, where the final aromatic reaction product cannot easily undergo hydrogenation in the presence of the metal hydride intermediate. If no such heteroaromatic product is formed, hydrogenation of the formed imine species can easily occur, resulting in *N*-alkylated amines. This approach has been successfully applied in the functionalization of anilines, using base metal catalysts. As such, several first row transition metal catalysts bound to triazine-based PN^3^P-ligands (*e.g*., Co, Fe, Cr, and Mn) can conduct this transformation successfully, usually requiring 1–3 mol % catalyst loadings.^76^ In some cases (Mn- and Cr-based systems, **59** and **61**),^76d,76e^ the amount of base required could be reduced to substoichiometric amounts. A more detailed mechanistic study on the manganese-mediated N-alkylation reaction revealed the importance of noncovalent interactions in lowering the activation barrier for the imine hydrogenation step.^74^^d^ Namely, upon deprotonation of the acidic NH groups, the potassium cation remains associated with the resulting anion through imine and triazine N···K interactions, which is supported by crystallographic studies (**59**, Figure 14). Similar interactions were also observed in reduced pyrimidine-diimine iron carbonyl complexes.^53^ For **59**, the potassium cation can further engage in dipole interactions with the incoming imine substrate, stabilizing the hydrogen transfer transition state.

The N-alkylation methodology based on “hydrogen borrowing” could also be extended to the C-alkylation of esters, amides, and alcohols.^77,78^ Cobalt catalysts based on triazine-chelates (**60**, Figure 13) were found to be remarkably active in ester and amide alkylation,^77^ using a large variety of aliphatic alcohols. In contrast, the analogous catalysts based on pyridine cores displayed only modest catalytic activities for the alkylation of amides and no catalytic activity for the alkylation of esters, under the same reaction conditions. An even more challenging transformation represents the β-methylation of alcohols, which could be achieved using cobalt (**60**) and manganese (**59**) catalysts.^78^ Interestingly, cobalt catalysts (**60**) are active if longer chained alcohols are employed as alkylating agents,^78a^ whereas in the case of methanol, only the manganese congener (**59**) is active.^78b^ Similar to ester and amide alkylation, the analogous catalysts based on pyridines remain inactive or perform significantly more poorly.

### Ketone Synthesis via Dehydrogenation and Ketone Hydrogenation Methods

The dehydrogenation of benzylic alcohols to give aldehydes has been recently engineered to allow the synthesis of 1,3-diketones from esters (Figure 13). The mechanism relies on an elegant combination of a base-mediated transesterification, followed by a metal-catalyzed dehydrogenation of the resulting phenoxide to the corresponding benzylketone, which engages a Claisen condensation with the resulting ester.^79^ The crucial dehydrogenation reaction is catalyzed by a triazine-based manganese catalyst (**59**).

Subjecting ketones to hydrogen pressure in the presence of manganese and cobalt catalysts (**58** or **60**) results in highly efficient synthesis of alcohols (Figure 13).^80^ For the cobalt-based catalyst, the triazine ring and the substituent in the para position (R = Me in **60**) played a crucial role in catalytic activity. Under the reaction conditions, the direct pyridine analogue performed significantly worse.^80a^ An even higher catalyst activity was observed for the manganese-based congener, requiring catalyst loadings below 0.5 mol % in most cases.^80b^

Cobalt catalysts based on triazine cores (**60**) have also been employed for dehydrogenative silylation and borylation of alkynes.^81^ Unlike the methodologies described above, the mechanism relies on alkyne and silane oxidative addition and reductive elimination sequences in a Co^I^ – Co^III^ redox cycle, rather than on chemical metal–ligand cooperativity. As such, for the dehydrocoupling reaction, no base is necessary and the catalytically active cobalt hydride species are generated via the activation of **60** with PhRSiH_2_. Nevertheless, even in this case, the electronic deficient nature of the triazine proved responsible for the high catalytic activity, with the pyridine analogues being less catalytically active. If HBPin is used as a reaction partner, competitive hydroboration of alkynes is observed. The fine-tuning of the R substituent in **60** determines whether the borane engages in dehydrocoupling (R = secondary amine) or in hydroboration of alkynes (R = alkyl or aryl).^81b^ If silanes are used as reaction partners, the method used for precatalyst activation was found to determine the reaction outcome: in the absence of an external base, dehydrocoupling is observed, while using LiO^t^Bu as activator stirs the reactivity toward hydrosilylation.^81c^ Derivatives of **60** with different variations of the R substituents on the heteroaromatic ring have been used for catalytic hydroboration and hydrosilyation of alkynes and olefins,^82^ or in the silylation of silanols.^83^

**Figure 15:**
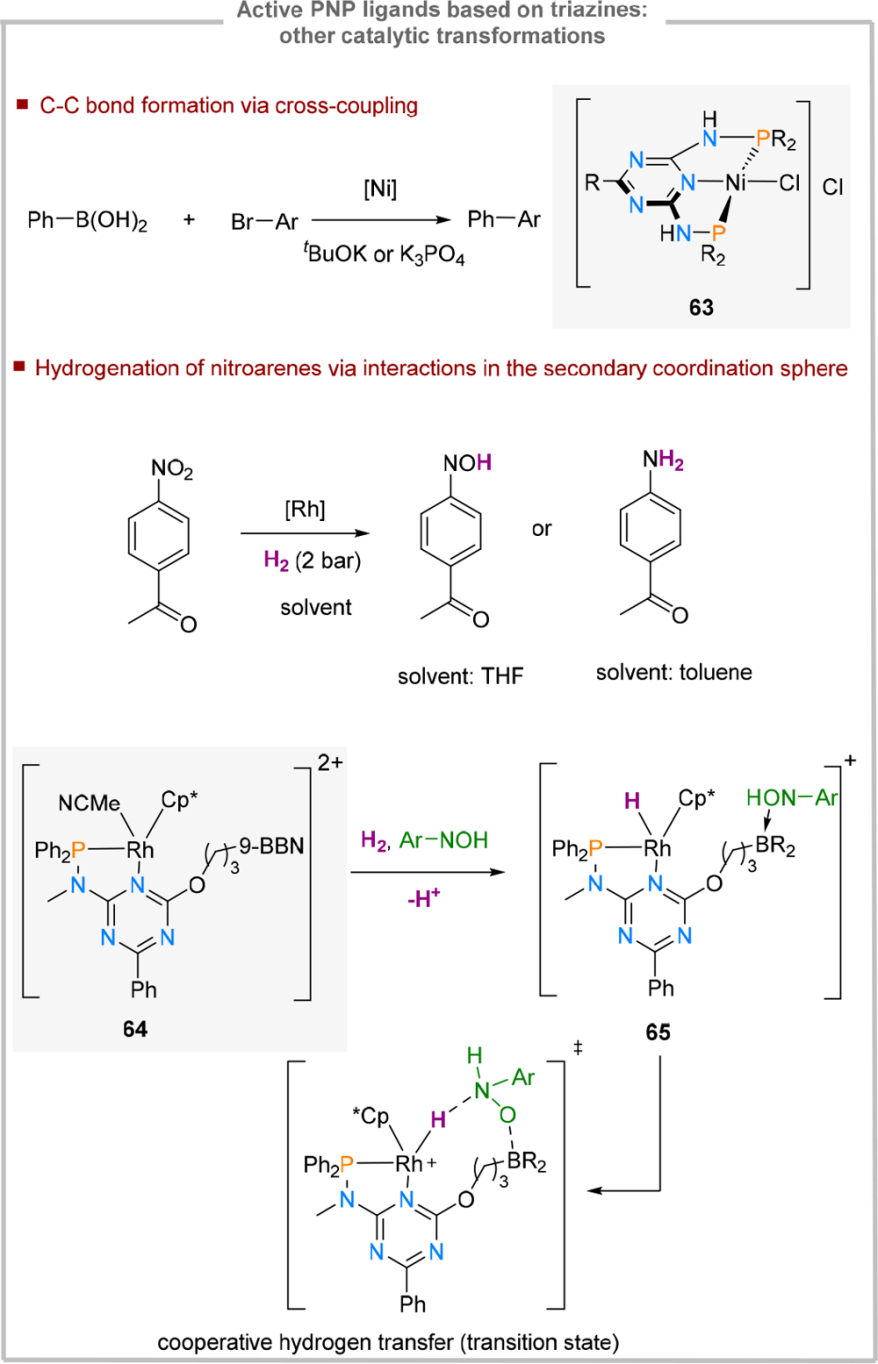
Active triazine-based PNP ligands.

The same triazine-based PN^3^P ligand has also been used in nickel mediated Suzuki cross-coupling using a wide range of alkyl and aryl bromides as coupling partners (**63**, Figure 15).^84^ The base is believed to play a dual role in this transformation. It is required for the activation of the boronic acid but also deprotonates the nickel-based catalyst. The authors postulate that the resulting deprotonated species are on-cycle intermediates.

A rhodium-based catalyst (**64**) for the selective hydrogenation of nitroarenes in the presence of ketones has also recently been reported.^85^ The ligand design relies on an electron-deficient triazine combined with electron rich phosphines and pentamethylcyclopentadienyl (Cp*) units, exerting a push–pull electron density mechanism. The triazine core is further functionalized with a flexible alkyl chain containing a Lewis-acidic boronic acid based on 9-BBN (9-borabicyclo[3.3.1]nonan). Mechanistic studies supported by computational modeling suggest that dihydrogen is activated in a heterolytic fashion to generate Rh–H (**65**). The pendant borane coordinates the substrate via the NO_2_ group, bringing it in the secondary coordination sphere of the metal center, which facilitates metal-to-substrate hydride transfer. The ligand design and the presence of a methylated N-linker between the phosphine and the triazine core makes this transformation mechanistically distinct from the ketone hydrogenation methodology mediated by **58** and **60** (Figure 13).

## Conclusions and Outlook

Controlling the steric bulk around the metal center is essential for lowering the activation barrier and kinetically blocking deactivation pathways. Challenges often arise when steric and electronic changes cannot be decoupled from one another, and therefore the respective effects cannot be studied separately. Therefore, modulating the electronic properties without affecting the steric environment around the metal center represents an attractive mechanistic tool. A convenient way to achieve this is by performing CH → N isosteric replacements on the supporting heterocycle. This overview attempted to demonstrate that diazine and triazine cores are attractive isosteric replacements for the ubiquitous pyridine cores, especially in the design of tridentate (pincer) ligands. We have shown cases where this approach can have significant advantages over the pyridine analogues. For example, enhancement of the core π-acidity can confer increased catalyst stability under reductive conditions or can modulate the hydricity of M–H units, affecting the rates of various hydrogen transfer reactions (*e.g*., C=O or C=C insertion, HAT). While the decreased electron density at diazine and triazine cores can destabilize metal–ligand interactions, this can be compensated by using phosphorus ligands in the flanking positions with respect to the metal center (*e.g*., PN^3^P-ligands). Another distinguishing feature of diazine ligands is the functionalization of the distal nitrogen atom (*i.e.*, remote-site functionalization via protonation or Lewis acid ligation), allowing further modulation of electron density and Lewis acidity of the metal center. Such Lewis acid–base interactions have also been observed in base-activated triazine-based ligands. The nitrogen atoms which are part of the heterocycle can anchor potassium cations which can further engage with basic functional groups on the incoming substrate, stabilizing the resulting transition state.

Nevertheless, despite the opportunities they can provide, diazines and triazines are significantly more seldomly employed in ligand design, especially when compared to their pyridine congeners. This can be traced to more synthetic difficulties in ligand design, the vulnerability of these heteroaromatic cores to nucleophilic attack (*e.g*,. under strong hydridic and alkylating conditions), and the sparseness of data on the electronic effects of diazines and triazines on catalytic activity. Therefore, an important challenge that remains to be addressed is the mapping of the electronic effects of polynitrogen-containing cores compared to their direct pyridine analogues, and the direct impact of these effects in catalysis. Moreover, the use of diazine- and triazine-based pincers in transition-metal-mediated enantioselective catalysis remains to be reported. Nevertheless, we believe that, with adequate ligand design and fine-tuning, diazines and triazines represent an attractive building block for catalyst design, which is already illustrated in the significant progress observed in the field over the past ten years.

## Data Availability

The data underlying this study are available in the published article.
